# Participant observation of time allocation, direct patient contact and simultaneous activities in hospital physicians

**DOI:** 10.1186/1472-6963-9-110

**Published:** 2009-06-29

**Authors:** Matthias Weigl, Andreas Müller, Andrea Zupanc, Peter Angerer

**Affiliations:** 1 Institute and Outpatient Clinic for Occupational, Social, and Environmental Medicine, Ludwig-Maximilians-University, Munich, Germany

## Abstract

**Background:**

Hospital physicians' time is a critical resource in medical care. Two aspects are of interest. First, the time spent in direct patient contact – a key principle of effective medical care. Second, simultaneous task performance ('multitasking') which may contribute to medical error, impaired safety behaviour, and stress. There is a call for instruments to assess these aspects. A preliminary study to gain insight into activity patterns, time allocation and simultaneous activities of hospital physicians was carried out. Therefore an observation instrument for time-motion-studies in hospital settings was developed and tested.

**Methods:**

35 participant observations of internists and surgeons of a German municipal 300-bed hospital were conducted. Complete day shifts of hospital physicians on wards, emergency ward, intensive care unit, and operating room were continuously observed. Assessed variables of interest were time allocation, share of direct patient contact, and simultaneous activities. Inter-rater agreement of Kappa = .71 points to good reliability of the instrument.

**Results:**

Hospital physicians spent 25.5% of their time at work in direct contact with patients. Most time was allocated to documentation and conversation with colleagues and nursing staff. Physicians performed parallel simultaneous activities for 17–20% of their work time. Communication with patients, documentation, and conversation with colleagues and nursing staff were the most frequently observed simultaneous activities. Applying logit-linear analyses, specific primary activities increase the probability of particular simultaneous activities.

**Conclusion:**

Patient-related working time in hospitals is limited. The potential detrimental effects of frequently observed simultaneous activities on performance outcomes need further consideration.

## Background

Hospital physicians' time is a valuable resource. Which activities clinicians allocate their time to is crucial to the quality of service. Two aspects seem to be especially important in the delivery of medical care: direct physician-patient contact and the burden of simultaneous task performance.

An effective physician-patient contact characterized by competent communication and compassion has repeatedly been identified as being one of the core principles of medical care [e.g. [1]]. More specifically, physicians in direct interaction with patients and with sufficient time are able to respond appropriately to patients' needs and concerns [2]. Also, physicians' work satisfaction relates to the time they have for patient interaction [3]. Organizational factors, e.g. those that contribute to tight time schedules, affect the nature and length of the physician-patient interaction [4]. The actual time spent in face-to-face contact has been shown to be limited. U.S. hospital physicians in internal medicine tend to work more time in indirect (56%) than in direct patient care (14%) [5]. Emergency physicians spend almost half of their time in indirect patient care [6]. Surveys in the U.S. showed that face-to-face interaction time with patients is about 55% of the entire working time in ambulatory settings and that the requirements of patient-related work outside the examination room are increasing [cf. [7]]. Physician work in hospital also involves a great deal of multidisciplinary communication and coordination of care [8]. In the U.S., up to 24% of working time is dedicated to communication activities [9], and in Australia a time share of 33% has been observed [10]. The share of documentation and charting activities is also of interest. In Germany, complaints are often voiced concerning "paperwork", documentation demands, or the allocation of enhanced administrative tasks [e.g. [11,12]].

Due to intensified work density, physicians often perform tasks simultaneously. Chisholm, Dornfeld et al. [13] showed that office-based primary care physicians, as well as emergency physicians, perform concurrent tasks in a substantial amount of their working time. Cognitive research shows that simultaneous tasks performance ("dual-tasks") is more prone to error or to reduced reaction times than sequential processing [14-16]. Subjects who are confronted with simultaneous action demands experience more overload in their work [e.g. [17]]. In work environments with high time pressures and high stress potential, e.g. in emergency departments, this may result in poor infection control behaviour, medical errors or the compromising of patient safety [16,18,19].

Due to the relevance of physician activities, we need a more accurate understanding of the type of tasks hospital physicians perform and the amount of time they devote to those tasks. First, there is a lack of standardized objective measures. The self-reports of physicians' time use that are frequently cited often differ in observed time periods [20]. Observation methods are empirically proven to be the most precise approach for assessing time and activity patterns [21,22]. Second, there is a lack in full shift observations. [10]. To reduce selection bias and error rate, a sufficient coverage of working time is important for participant observations [22]. Only a few studies take advantage of entire day shifts [10,20]. Most studies have used observation period lasting between 180 min [6] to 3–5 hours [9]. Existing 24h-observations have seldom been undertaken with physicians as subjects [e.g. [23,24]]. Furthermore, to assure a careful and reliable measurement, examinations of consistency indicated by inter-rater agreements are needed [25].

In this preliminary study we continuously observed complete day shifts of German hospital physicians using a classification system of physicians' activities. The following series of questions are addressed: (1a) What activities do hospital physicians allocate their time to in surgical and internal medicine specialties?; (1b) Do hospital physicians perform different activities in different clinical units (wards, emergency ward, intensive care, operating room)?; (2) How much time do physicians spend in direct patient contact?; (3a) How much time do hospital physicians spend performing simultaneous activities?; (3b) What specific combinations of simultaneous activities do hospital physicians have to perform?

## Methods

This paper conducted participant observations of hospital physicians' activities in a municipal 300-bed teaching hospital in Southern Germany (circa 31,000 patients/year; 49% ambulatory). It is maintained in public ownership, employs 99 physicians and can be regarded as a typical German hospital, which has on average 243 beds and is publicly owned and run [26].

Participant observations pose a potential conflict between the researcher's pursuit of data and ethical considerations [27,28]. In accordance with research ethics (e.g. the Helsinki Declaration) there is an area of conflict around maintaining professional confidentiality, the principle of voluntariness, and the participation in medical care interactions. The study only focused on physicians' activities and no patient-related information was identified, assessed, or recorded. Where possible patients were informed beforehand about the aim of the presence of observers. Observers left the room for confidentiality purposes when requested. However, in the hospital setting for patients in some clinical units it was not always practically possible to avoid observing situations that involve patients who had not given their consent (e.g. in the emergency ward or intensive care unit). Therefore both observers were strictly obliged to respect confidentiality and bound to follow medical ethical restrictions. Information of observed physicians was kept both anonymous and confidential. This project was approved by the Ethics Committee of our clinic and the department directors at the study site.

### Participants

Inclusion criteria were that hospital physicians had to be employed full-time and had to have sufficient tenure within the hospital (minimal four months). Only physicians either currently undergoing or with finished post-graduate training for a specialty certification were selected. To cover a consistent set of functions senior or head physicians were excluded. All eligible physicians (N = 32) of the four departments were informed beforehand about the study. Participation in the study was voluntary, and verbal or written consent was obtained before the observation. Two physicians refused to participate. The dates of the observations were selected randomly.

Finally, 23 physicians from two specialties, namely, internal medicine (INT, N = 13) and surgical medicine (SURG, N = 10), participated in the study. 12 physicians were observed twice. Seven physicians were not on duty within the selected shifts. The share of female doctors varied between 40% (SURG) and 61.5% (INT). The average tenure with the present hospital was 7.0 years (SD = 4.6) for the internists and 7.4 years (SD = 6.1) for the surgeons. The percentage of doctors with a specialist certification was 20% (SURG) and 38.5% (INT). Only day shifts during the week were selected because only then physicians are clearly assigned to a clinical unit (which is important for question 1b); in all other type of shifts physicians are required to work across departments and wards. 12-hour shifts are scheduled in the intensive care unit. In all other clinical units the physicians are obliged to work 8.5 h day shifts. In the examined hospital, internists partially staff the interdisciplinary intensive care unit as well as the emergency ward.

### Observation instrument: Classifying physicians' activities

The observational time motion study can be considered as the 'gold standard' of time measurement [[21], p. 374]. Participant observations to record physicians' activities are shown to be a valid and reliable approach to assess clinicians' time allocation [20,21]. As one comparative study showed this method is more accurate than alternative methods (e.g. self-administered time logs, interviews) [21].

Few *activity classifications for hospital physicians' activities *could be found in literature, and these differed in structure and applicability. The present category systems vary according to the number of activities, disciplines, or specialties [22,29-31]. Thus, we developed an interdisciplinary and applicable observation instrument to classify physician activities: First, a list of hospital physician activities was compiled by the authors. We reviewed existing categories and adopted the distinction of direct, indirect patient care and personal activities [6,13]. Secondly, we discussed it with physicians from different specialty areas. Thirdly, six pilot observations were performed to test the instrument's applicability, the full coverage of occurring physician activities, as well as to specify classification criteria. Problems in handling and scoring discrepancies were discussed. The results of these developmental observations will not be reported within this paper. The final classification system of hospital physicians' activities consists of 36 distinct task categories (see table 1). Generally, four major categories and 11 subcategories were assessed: direct patient contact, all activities performed face to face with patients (e.g. diagnostics, therapy); indirect patient contact, all patient related activities when the patient is not at hand (e.g. documentation, conversation with staff) other professional activities (e.g. teaching, research) and personal activities (e.g. regular breaks, sleeping).

**Table 1:** Categorization of hospital physicians' activities

*Name of Category*	*Activity*
**Direct patient contact**	

Patient Communication	1 Patient Communication (regularly, scheduled, ward round)
	
	2 Patient Communication (irregular, due to patient interruption)

Diagnostics	3 Diagnostic I: physical examination of the patient
	
	4 Diagnostic II: blood withdrawal
	
	5 Diagnostic III: machine aided examinations in functional departments

Therapy	6 Therapy I: Drug treatment
	
	7 Therapy II: Physical – non invasive – treatment
	
	8 Therapy III: Invasive treatment
	
	9 Therapy IV: Mixed emergency treatments
	
	10 Therapy V: Surveillance of patients in critical conditions and situations

Consultancy	11 Consultancy service for other departments
	
	12 Examinations for medical opinions

**Indirect patient contact**	

Documentation	13 Documentation/Charting
	
	14 Activity documentation (DRG Coding)

Conversation with staff	15 Conversation with colleagues (patient related)
	
	16 Conversation with nursing staff
	
	17 Conversation with assistance personnel
	
	19 Telephone conversation

Conversation others	18 Conversation with relatives
	
	20 Conversation with Others

Organizing	21 Organization/Work Flow
	
	22 Transfer/Walking times
	
	23 Arranging and ordering things

Meetings	24 Regular meetings with nursing staff
	
	25 Regular meetings with senior and head physician
	
	26 Clinic conference
	
	27 Interdisciplinary conferences
	
	28 Reviews of findings in the department
	
	29 On call in the hospital

**Other professional activities**	30 Teaching/Instruction
	
	31 Supervision of/by colleagues
	
	32 Research
	
	33 Training/education
	
	34 Reading (Training purpose)

**Personal activities**	35 Personal time/Regular Breaks
	
	36 Resting/Sleeping

Note: Definition of activities is not provided.

A *simultaneous task performance *was coded when two activities were obviously performed in a timely concurrent manner [13,32]. An obvious overlay of concurrent activities was the core criterion [33], e.g. a physician writing documentation while talking to a colleague. During a parallel performance, we distinguished between primary and secondary activities. Primary activities were ongoing activities. When another activity was started concurrently before completing the first, it was deemed 'secondary'.

We performed an antecedent test of the instrument's reliability: six physicians (3 surgeons, 3 internists) were observed by two raters simultaneously. One rater was a work psychologist with experience in this type of assessment; the second rater was a medical student who received instruction and training beforehand. To control for bias the observers were forbidden from discussing their ratings and neither one had insight into the records. The sample observation periods covered in sum 291.5 minutes (Range: 34.5 to 69 min). The Kappa-coefficient indicating inter-rater agreement was .71 (T = 41.6; p = .00). This is regarded as substantial agreement and points towards good reliability [34].

The same two raters carried out the participant observations of the main study. They "shadowed" the physicians throughout the entire day shift, and made paper-based records of the type and duration of activities. In order to minimize observational effects, physicians were surveyed at a respectable distance [cf. [6]]. Both observers were instructed not to interrupt physicians and were dressed like interns.

### Study design and data collection

Participant observations to assess physicians' activities and time allocation were applied. Thirty-five day shift assessments were conducted, with an overall duration of 303.1 hrs (18,184.8 min). The observed shifts had an average duration of 8 hrs 39 min (SD: 1 hr 42 min; Range: 4 hrs 54 min – 14 hrs 05 min). Table 2 presents the number and average lengths of observations.

**Table 2:** Study design and duration of observations

**Specialty**	**Clinical units**	**No of observations**	**Mean Duration **(SD)
Surgery	Ward (4 wards)	8	8:43:14 (0:25:20)
	
	Emergency Ward	6	8:03:44 (2:37:53)
	
	Operating room	3	8:28:49 (0:02:41)
	
	Sum	17	143:34:50

Internal Medicine	Ward (4 wards)	12	8:24:56 (0:34:35)
	
	Emergency Ward	3	7:21:06 (2:09:38)
	
	Intensive Care Unit	3	12:10:22 (1:42:52)
	
	Sum	18	159:33:47

Note: Time [hh:mm:ss]; SD Standard Deviation

### Data entry and statistical analyses

Recorded data were kept anonymous and confidential. The data was checked for correctness and implausible values. The analysis was performed using SPSS 15.0 for Windows. We used the Mann-Whitney U Test for group difference tests due to the small group sizes and the non-parametric character of the data. Because of the exploratory nature of our study, no multiplicity adjustment was applied. For testing the temporal duration of the activities, T-Tests were applied. To answer the third research question – regarding combinations of simultaneous activities – we examined whether certain parallel activities are more likely under the condition of particular primary activities. In statistical terms, we compared the unconditional probability that a parallel activity will occur at any time with the conditional probability of this activity occurring at the same time as primary activity. To test for the statistical significance of unconditional probabilities, we used logit-loglinear models [35,36]. Logit-loglinear models are χ^2^-tests that allow for an examination of the relationship between nominally-scaled dependent and independent variables.

## Results

Overall, 1,757 surgeons' activities were observed [in 143:34:50, (hh:mm:ss)], compared to 2,493 internists' activities (158:48:06).

### (1a) What activities do hospital physicians allocate their time to in surgical and internal medicine specialties?

Physicians from both specialties spend a large share of their working time on documentation and charting, as well as on conversation with staff (colleagues and nurses). Documentation was found to be the most frequent activity for both groups (SURG 25.7%; INT 32.0%). Surgeons were also observed carrying out frequent invasive treatments (18.8%) and having conversations with colleagues (8.4%). Frequent activities for internal medicine physicians were communication to patients (9.3%) and phone calls (8.8%).

Specialty differences regarding the categorized activities can be found in Table 3. Internal medicine physicians tend to spend less time in direct patient contact (Z = -1.73; p = .08). Whereas surgeons were observed to allocate less time to activities categorized as indirect patient care (Z = -1.91, p = .06). This is due to the larger amount of time that surgeons spent in therapy, i.e. operations or other invasive treatment.

**Table 3:** Time proportion of categorized activities of surgical and internal medicine physicians during day shifts

	**Surgeons**	**Internists**	**Test of group differences (%t)**		
	
	**%t**	**%t**	**MW-U**	**Z**	**p**
**Direct patient contact**	34.1	20.9	100.5	-1.73	.08

Patient communication	8.0	9.8	101.0	-1.72	.09
Diagnostics	5.8	7.7	117.0	-1.19	.23
Therapy	20.3	3.4	96.0	-1.90	.06

**Indirect patient contact**	56.5	69.4	95.0	-1.91	.06

Documentation	26.8	33.1	103.0	-1.65	.10
Conversation with staff	16.9	21.1	98.0	-1.82	.07
Conversation with others	1.6	2.6	116.0	-1.23	.22
Organizing	1.7	2.9	95.5	-1.91	.06
Meetings	9.5	9.7	146.0	-0.23	.82

**Other professional activities**	.3	1.4	128.0	-1.36	.18

**Personal activities**	9.1	8.3	141.5	-.38	.70

Note: %t percentage of time spent in observed activity in regard to the overall observation time; significance test (%t): Mann-Whitney U-Test for independent samples (p-two sided significance)

Regarding the temporal duration of the single activity sequences, the average length of documentation periods yielded no specialty difference [SURG 04:36 (min:sec); INT 04:23; df = 1173; T = .69; p = .49]. Internists performed longer communication intervals with patients (SURG 02:48; INT 03:21; df = 507; T = -2.07; p = .04). Conversation with fellow colleagues (SURG 03:37; INT 03:17; df = 414; T = .8; p = .43), as well as talking on the phone (SURG 01:55; INT: 02:06; df = 592; T = -1.09; p = .28), showed no specialty differences.

### (1b) Do hospital physicians perform different activities in different clinical units?

A total of 2,324 activities in all wards were coded (in 169:59:35). On the emergency ward, the physicians were monitored on 1,206 activity sequences (70:24:38), in the intensive care unit on 599 activities (36:31:06), and in the operating room on 121 activity sequences (25:26:29). Table 4 presents the observed temporal share of the categorized activities.

**Table 4:** Temporal proportion of physicians' activities on the clinical ward, emergency ward, in the intensive care unit, and in the operating room

	**Ward**	**Emergency Ward**	**Intensive Care Unit**	**Operating room**
	
	**N = 20**	**N = 9**	**N = 3**	**N = 3**
**Direct patient contact**	19.5	33.1	18.7	74.6

Patient communication	9.7	12.9	2.5	2.8
Diagnostics	4.9	13.7	6.9	.4
Therapy	4.9	6.5	9.2	71.5

**Indirect patient contact**	70.8	56.4	72.8	18.1

Documentation	35.0	29.2	27.8	3.1
Conversation with staff	19.0	18.2	30.0	7.1
Conversation with others	2.4	1.4	3.6	.1
Organizing	2.6	1.6	1.6	3.3
Meetings	11.8	6.1	9.8	4.6

**Other professional activities**	1.3	.6		

**Personal activities**	8.4	9.9	8.5	7.2

Note: N Number of observations; numbers in cells: %t percentage of shift's time spent in observed activity.

Due to the exploratory character of this examination, as well as to the low cell frequencies, no significance tests for unit or specialty differences were carried out. Physicians on clinical wards performed documentation and charting tasks for almost one third of their time, which is slightly higher than on the emergency ward and in the intensive care unit.

### (2) How much time do hospital physicians spend in direct patient contact?

Overall, we found physicians work in direct patient contact for 25.5% of their day shift. Almost significant differences between the specialties in both major categories were observed (see Table 3). Surgeons tended to work more frequently in direct patient contact; internists were observed spending more time in indirect patient contact.

Regarding the temporal share of direct and indirect patient activities in different clinical units, physicians in operating rooms and on the emergency ward were more frequently working in direct patient contact (see table 4). On the intensive care unit and the clinical ward physicians spent a large share of time in indirect patient contact.

### (3a) How much of the time do hospital physicians spend performing simultaneous activities?

Regarding the temporal proportion, we found surgeons performed parallel simultaneous activities for 20.3% and internists for 17.3% of their working time. This difference was not significant (U = 128.0; Z = -.83; p = 0.41).

Regarding the four clinical units a high degree of simultaneous task performance was in particular observed on the emergency ward (30.6%). On clinical wards (16.6%) and in the intensive care unit (17.1%), fewer simultaneous activities were noted.

### (3b) What specific combinations of simultaneous activities do hospital physicians have to perform?

To obtain an adequate level of categorization and sufficient cell frequencies, we classified the activities into seven subcategories (see Table 5). All events of simultaneous task performance were taken into account (N = 750). The most frequent activity combinations were documentation and simultaneous conversation with colleagues or staff (16.7%), and talking to colleagues or staff and doing documentation (12.7%). Communication with patients (25%), documentation (26%) and conversation with colleagues (30%) were the most frequent simultaneous activities.

**Table 5:** Conditional probabilities of observed parallel and its primary activities (logit-linear analysis; N = 750)

	**Parallel activity**							
	
Primary activity	**1**	**2**	**3**	**4**	**5**	**6**	**7**	**Unconditional probability**
1. Communication patient	.00	**.27**	.01	**.48**	.13	**.09**	.01	.22
2. Diagnostic	**.67**	.00	.02	.04	.22	.02	.04	.15
3. Therapy	.26	.00	.00	.02	**.60**	.04	.09	.06
4. Documentation	**.37**	.00	.00	.00	**.56**	.01	.05	.30
5. Conversation (colleagues, nurses)	.10	.02	**.10**	**.77**	.11	.04	.08	.20
6. Conversation (relatives & others)	**.57**	.00	.00	.36	.07	.00	.00	.02
7. Else	.00	.03	.02	.11	.18	.00	**.26**	.05

Unconditional probability	.25	.07	.02	.26	.30	.04	.06	1

Note: N = 750; bold probabilities p < .01.

Secondly, we explored which parallel activity is more likely under the condition of a certain primary activity (see Table 5):

The probabilities are interpreted in columns representing the likelihood for a certain secondary activity under the condition of a primary task. The accumulated probabilities in the last row represent the overall unconditional probability that a specific parallel activity is carried out simultaneously to any other primary activity. Shaded probabilities point out that the occurrence of a certain parallel activity becomes more likely when a specific primary activity is performed. For instance, the unconditional probability of communicating with patients parallel to any other primary activity is 25%. But, under the condition of diagnostics, the probability of communicating with patients increases significantly, up to 67%. Similarly, the probability of communicating with patients also increases under the condition of documentation (37%) and the condition of conversation with others (57%). Altogether, the results show that the performance of specific primary physician activities increases the probability of specific parallel activities.

## Discussion

The present findings support the view that hospital physicians dedicate limited working time to direct patient interaction [cf. [9]]. Physicians spent most of their time in activities involving indirect patient contact, especially due to charting and documentation demands [11]. Although the importance of direct patient conversation has been emphasized, the present findings show that physicians engage in relatively short communication episodes.

Regarding the activities in the examined units, we found that hospital physicians in the operating room and emergency ward tend to spend more time in direct patient interactions [cf. [6]]. On clinical wards, we found similar activity patterns to those found in US hospitals: most time was spent in documentation and communication activities with staff and only a fifth of it was dedicated to direct patient care [9]. Professional conversation with colleagues and nurses was observed in particular in the intensive care unit (30% of the time), reflecting the high communication demands in intensive care and the tight interplay between the disciplines [37].

Concerning simultaneous activities, we found that physicians were carrying out concurrent activities for almost a fifth of the working time [cf. [9,10]]. On the emergency ward, physicians were even engaged in simultaneous activities for almost a third of the time, which indicates the high workload and error potential in emergency care [18,38]. Concerning the character of the secondary activities, documentation was most often performed as a concurrent task. Regarding simultaneous activity combinations, we often observed documentation and simultaneous communication with colleagues or staff or, conversely, conversation with co-workers and concurrent documentation tasks. When physicians were engaged in diagnostics, in documentation, or in conversations with others (e.g. relatives), the probability of parallel patient conversations was found to be significantly higher. Similarly, concurrent documentation was significantly related to patient communication and conversation with colleagues or staff.

The methodological strengths of the present study are the observational assessment of physicians' activities, the preliminary test of the instrument's reliability, and the overall duration of observations. Furthermore, the use of full-shift observations allows for a detailed insight into activity patterns throughout the entire daily working time. To reduce the error rate, the large amount of observed working time is considered crucial for time motion approaches [22]. Additionally, the assessment of simultaneous activities provides a methodology for assessing the degree of competing demands and task loads in physician activities [39]. The present instrument, with its specific and broader activity categories, may be applied in several specialty areas. It can therefore be used for activity recordings, as well as for workplace analyses or evaluation purposes.

### Limitations of the study

This preliminary study should be interpreted in light of several limitations and sources of potential error. To begin with, the data is not a full representative reflection of what hospital physicians generally do. We assume that the extended observation time is sufficient enough to provide an accurate insight into the normal daily routine work of hospital physicians in Germany. But the convenience sample limits the external validity of the findings. Due to practical constraints, we did not observe internal medicine physicians in functional diagnostic units (e.g. cardiac catheterization, endoscopy). During the time of observation almost no medical students or interns were on site. So we hardly observed physicians with supervisory or teaching obligations. Yet it is known that doctors in teaching hospitals are much more occupied with supervising activities [cf. [5]]. This may also have an effect on the nature and duration of a physician's activities as well as the amount of additional (simultaneous) work-load (e.g. performing patient diagnostics and simultaneously instructing a resident). Constraints imposed by the particular work organization, or particular clinical procedures, as well as by personal experiences and routine levels, may impact the activity patterns in an essential way, as well. In this study only day shifts were observed. Particularly in emergency settings, as well as during night or weekend shifts, a very different picture of physician activities may arise. Although we took various measures to avoid influence, observer presence may impair the data: the presence of third parties might detrimentally change physicians' and patients' behaviour (e.g. decreased tendency to take breaks or reduced intimate questions by patients). This potentially altered behaviour (a.k.a. Hawthorne effect) has to be taken into account when considering the validity of the present results.

Recording multiple concurrent events, especially in complex work environments, poses a difficult challenge in observation studies [40], thus demanding the use of validation studies using other data sources, as well.

Although the study only focuses on physician-related information, we acknowledge ethical concerns regarding 'third party privacy and consensus' during observations [27,28]. Within a hospital setting it is not always practicably possible to avoid observing situations that involve patients who had not given their consent [28]. Although we handled this issue carefully, bound all observers to confidentiality and medical restrictions, did not assess or record any patient related data, we are aware of the potential challenges of this approach [27,28]

### Recommendations for further studies

Further research attempts should focus on theoretical, as well as methodological, considerations. Theoretically, investigations in hospital physician work may call into question what can be considered a reasonable amount of time to spend in direct patient interaction. Confounding factors, such as specialty, proficiency, patient characteristics, or work assignments, may play a role, as well as strategic and economic issues.

Further, the potential effects of simultaneous activities on information loss or medical errors needs to be examined empirically [9]. A distinction needs to be made between non-problematic and problematic task-combinations (e.g. simultaneously prescribing medication). The latter cause increased cognitive complexity and may lead to medical errors; e.g. attention slips or diverted concentration leading to prescribing errors [41,42]. Thus, investigations regarding the detrimental effects of simultaneous tasks on various outcome variables are needed.

In our study we did not take into account the role of electronic records. An elaborated implementation of electronic assistance may be useful but must be carefully considered regarding the anticipated outcomes. A review shows that the use of electronic records does not necessarily reduce documentation time [43], but other research suggests that it might lead to slight increases in time spent directly with patients [31].

Methodological considerations should focus on the reliability and validity of the present instrument. Although participant observations are shown to be a valid approach to assess physicians' time allocation [21] this approach stresses time, human, and attention resources. The use of alternative assessments may be more warranted in other settings or under different conditions. An advanced adaptation to various hospital contexts may further improve the instruments applicability.

Finally, intervention or work design approaches may incorporate the study results. For instance, an elaborated implementation of electronic assistance may be advisable. But it must be carefully considered regarding the anticipated outcomes.

## Conclusion

This study offers several findings with respect to the allocation of hospital physicians' time – a critical resource in health care. We found that a limited share of time is allocated to direct patient contact, but much more time is spent in documentation and charting. Further, the demand of simultaneous task performance is prevalent to about a fifth of the entire working time.

This study's principal findings of clinicians' time allocation allow us to draw conclusions especially relevant for work redesign approaches in hospital physician work. An identification of activities with higher patient-care value, as well as the elimination or delegation of administrative or documentation tasks might be a first step to expand patient-related working time [5]. Work redesign attempts may also focus on frequent communication processes within and across disciplines. Further approaches may challenge the temporal fragmentation of physicians' activity patterns to establish continuous workflows [24]. Arranging for continuous information flows, adequate electronic assistance, and a low level of interruptions may facilitate the burden of multitasking and thereby improve patient safety [16,44].

## Competing interests

The authors declare that they have no competing interests. There are no financial or other conflicts to report.

## Authors' contributions

MW conceived the idea and was primarily responsible for study planning and design, planning, coordination, supervision of the literature review, analysis of results and manuscript preparation and revision. AM contributed to study design and collaborated in analysis and interpretation of results and manuscript preparation. AZ collaborated in study design, coordinated the running participant observations and manuscript preparation. PA supervised study planning and design, data acquisition, analysis of results and helped draft the manuscript. All authors made a critical review of the manuscript. All contributors approved this version submitted for publication to the BMC Health Services Research.

## Pre-publication history

The pre-publication history for this paper can be accessed here:


